# Outlines of Pathology and Practice of Medicine

**Published:** 1843-04

**Authors:** 


### ART. XXIII.

1. *Outlines of Pathology and Practice of Medicine.* By W. P. ALISON, M.D. F.R.S.E., Professor of the Practice of Medicine in the University of Edinburgh. Parts I. II.—*Preliminary Observations: Inflammatory and Febrile Diseases.—Edinburgh,* 1843. 8vo, pp. 499.

2. *Methodus Medendi; or the Description and Treatment of the principal Diseases incident to the Human Frame.* By H. M'CORMAC, M.D. Professor of Medicine in the Royal Belfast Institution.—*London,* 1842. 8vo, pp. 574.

ALTHOUGH the work of Dr. Alison is intended as a text-book for students attending his lectures, it is calculated to fulfil much greater objects, being, in fact, according to its extent, the best digested and most philosophical manual of the practice of medicine which has appeared for many years,—we were almost going to say, since the publication of the admirable "First Lines" of Dr. Alison's greatest predecessor. If there is one man in the profession better qualified than any other to write such a work, we think Dr. Alison is that man. He has been long acknowledged by all who know him to be in his character a pattern of a scientific physician; and his opportunities—we may say the necessities of his position—have been such as to improve his natural capacities to the utmost. The public offices which he has so long filled have demanded and fostered alike scientific knowledge and practical skill. The consequence is, that, to an exact and extensive acquaintance with what has been discovered and done by others in all branches of medicine, Dr. Alison adds the skill and tact which can only be acquired by long and diligent attention to the treatment of disease in an extensive field of practice. It would, however, be doing injustice to its author to regard the present work as intended to convey to the profession a full view of the results of his medical experience: it ought rather to be viewed as a concession to the necessities of his pupils; or, at most, as a specimen of the treasures yet in store for us. The nature of the volume is justly expressed in the preface:

"In attempting to compress, within the limits of a text-book for lectures, the facts which seem to me best ascertained in regard to the nature, progress, and symptoms of diseases, and the effects of remedies upon them, it has been my object to simplify, as far as possible, both the diagnostic marks of diseases, and the practical rules for their treatment, dwelling only upon those, an accurate knowledge of which may be acquired without much difficulty, and on which it has appeared to me, in practice, that we can rely with most confidence. I have endeavoured to connect these practical rules and directions with as full a statement as the limits of such a work will permit, of the grounds of those opinions, in regard to the causes, the intimate nature, and fatal tendency of diseases, which seem to me, in the present state of our knowledge, to be supported by the best evidence; because, notwithstanding all that has been said, and may be said, against medical speculation, I am fnlly convinced of the truth of the observation of Dr. Cullen, that 'at all times the practice of medicine has been, and still is, with every person, founded more or less upon certain principles established by reasoning;' from which it evidently follows, that any one who undertakes to teach the practice of medicine must be prepared to explain the grounds of his opinions; as well as to state the facts, and describe the appearances, on which he is to found his practical precepts."

Everywhere the work exhibits the author's exact information as to the discoveries of others, his just appreciation of contending claims, his cool judgment in weighing the truth, and his own practical knowledge of the treatment of the diseases. There is a cautious sobriety pervading it which commends itself to those who have experienced, in sick rooms, the difficulty and uncertainty of our art, and which cannot fail to be valuable to younger minds whose zeal and hope are apt to carry them beyond the narrow bounds which limit our capabilities; it will help to teach them beforehand, a lesson which they must otherwise learn by experience, that in the art of medicine, the meed of successful practice is rarely won by genius or learning alone, and almost always by sound common sense, a cool and sober judgment, and diligent observation. The chapter on the Treatment of Fever is admirably characteristic of the author's knowledge and discretion.

The *Methodus Medendi* is the very antithesis of Dr. Alison's work. Dr. M'Cormac has a huge appetite, but not a corresponding digestion. He must be a vast reader, a very *helluo librorum*—but, notwithstanding his title, without *method.* Anything like order or arrangement hardly exists; one fact, or opinion, or observation, follows another in rapid succession, with the loosest possible connexion; the author appearing to delight in giving his mind the free rein with little check as to speed or direction. And yet the book is a full book and a learned book. You cannot read a chapter of it and doubt that Dr. M'Cormac is thoroughly aware of all that has been written upon the subject of it; and you see that he gives in short compass the condensed result of his multifarious reading: but there is chaff as well as grain, and the grain is nowhere winnowed clean. The author has not sufficiently selected, nor weighed, nor judged the mass of facts and opinions he has collected. We cannot therefore recommend the *Methodus Medendi* to the student or young practitioner; they would be constantly puzzled and perplexed by it, if not positively led astray. But the work of Dr. M'Cormac will be most acceptable and valuable to that numerous class of readers, who have at one time been diligent students, and who would often, when a difficult case puzzles them, willingly open some book which could recall what they had forgotten, or give them a short statement of a mass of facts on which they may exercise their own reasoning and judgments; to them, accordingly, we recommend it.

